# Yet Another Automated Gleason Grading System (YAAGGS) by weakly supervised deep learning

**DOI:** 10.1038/s41746-021-00469-6

**Published:** 2021-06-14

**Authors:** Yechan Mun, Inyoung Paik, Su-Jin Shin, Tae-Yeong Kwak, Hyeyoon Chang

**Affiliations:** 1 Deep Bio Inc., Seoul, South Korea; 2 Department of Pathology, Gangnam Severance Hospital, Yonsei University College of Medicine, Seoul, South Korea

**Keywords:** Pathology, Cancer imaging

## Abstract

The Gleason score contributes significantly in predicting prostate cancer outcomes and selecting the appropriate treatment option, which is affected by well-known inter-observer variations. We present a novel deep learning-based automated Gleason grading system that does not require extensive region-level manual annotations by experts and/or complex algorithms for the automatic generation of region-level annotations. A total of 6664 and 936 prostate needle biopsy single-core slides (689 and 99 cases) from two institutions were used for system discovery and validation, respectively. Pathological diagnoses were converted into grade groups and used as the reference standard. The grade group prediction accuracy of the system was 77.5% (95% confidence interval (CI): 72.3–82.7%), the Cohen’s kappa score (*κ*) was 0.650 (95% CI: 0.570–0.730), and the quadratic-weighted kappa score (*κ*_quad_) was 0.897 (95% CI: 0.815–0.979). When trained on 621 cases from one institution and validated on 167 cases from the other institution, the system’s accuracy reached 67.4% (95% CI: 63.2–71.6%), *κ* 0.553 (95% CI: 0.495–0.610), and the *κ*_quad_ 0.880 (95% CI: 0.822–0.938). In order to evaluate the impact of the proposed method, performance comparison with several baseline methods was also performed. While limited by case volume and a few more factors, the results of this study can contribute to the potential development of an artificial intelligence system to diagnose other cancers without extensive region-level annotations.

## Introduction

Prostate cancer is the second most common malignancy in men worldwide and the second leading cause of cancer death among men in the United States.^1^ Prostate cancer prognosis depends on numerous factors, including histologic grade, type, cancer stage, and patient condition.^2^

A prostate needle biopsy is the most reliable diagnostic method performed on patients suspected to have prostate cancer.^3^ The Gleason score, which is assigned after the prostate needle biopsy by a pathologist after examining the tissue under a microscope, is the most powerful prognostic predictor and provides the basis for selecting a treatment modality.^4^

The Gleason grading system was devised in the late 1960s by Dr. Donald F. Gleason and members of the Veterans Administration Cooperative Urological Research Group.^5^ The Gleason grading system categorizes architectural features of tumors using a five-point scale, designating patterns 1 and 5 as the most differentiated and the least differentiated type of cancer, respectively. The Gleason score for a needle biopsy sample is the sum of primary and secondary pattern numbers, where the most prevalent pattern in the sample is graded as the primary and any amount of the worst pattern is graded as secondary. The Gleason grading system suffers from a well-known lack of interobserver reproducibility among practicing pathologists.^6,7^

A new classification for prognostic grade grouping was proposed in 2013 by a research group at the Johns Hopkins Hospital, resulting in five prognostically distinct grade groups, namely grade group 1 = Gleason score ≤ 6, grade group 2 = Gleason score 3 + 4 = 7, grade group 3 = Gleason score 4 + 3 = 7, grade group 4 = Gleason score 4 + 4 = 8, and grade group 5 = Gleason scores 9 and 10.^8^ The new grading system was accepted at the 2014 ISUP consensus conference, and the terminology of grade groups 1–5 has also been accepted by the World Health Organization in 2016.^9,10^

The computer-assisted analysis of images in the field of medical imaging is attracting increasing attention as a major research topic, driven by breakthroughs in artificial neural networks, often termed deep learning (DL), and a set of techniques and algorithms that enable computers to discover complicated patterns in large data sets.^11,12^

With advancements in the whole-slide image (WSI) technique and the Food Drug Administration approval for using a digital pathology system in primary diagnosis, computer-assisted analysis has been actively studied across all areas of pathology.^13^

Automated Gleason grading is an actively studied topic in the research of computer-assisted pathological diagnosis. Several outstanding results have recently been reported, whose grading performance is comparable to those of participating pathologists.^14–17^ They are commonly based on a two-stage architecture utilizing a DL model that separately recognizes Gleason patterns 3, 4, and 5 to extract features such as pattern-wise size and likelihood, which are then fed into the Gleason grade prediction model.

To this end, researchers have manually performed region-level Gleason pattern annotation tasks on WSIs,^14,15^ extracted diagnostic marker annotations on WSIs using computer vision techniques,^16^ or employed an epithelial tissue detection model, which was developed using immunohistochemistry-stained tissue slide images.^17^ All of these techniques involve large manual annotation costs and/or the development of complex algorithms.

Based on the assumption that the prostate cancer detection model actually learns the features that differentiate Gleason patterns, we developed a convolutional neural network (CNN)-based Gleason grading system named *Yet Another Automated Gleason Grading System* (YAAGGS) that accepts the WSI-level feature maps constructed using a CNN-based prostate cancer detection model that trained by slide-level annotations using the multiple-instance learning (MIL) method, and predicts the corresponding grade groups. We evaluated the prediction performance of the system on the datasets from two hospitals, Hanyang University Medical Center (HUMC) and Korea University Guro Hospital (KUGH), in the inter-institutional setting to analyze the generalization power of the model across the institutional boundary (training on HUMC and validating on KUGH), as well as in the holistic setting to assess its best achievable performance (training on a part of HUMC + KUGH and validating on the remainder). An additional experiment was performed in the holistic setting to measure the effect of the dataset size on the model performance. For the external validation, we applied our model on data from the Gleason 2019 Challenge^18,19^, which is publicly available.

The proposed system was also compared with several baseline methods. As the performance indices, the Cohen’s kappa score^20^ (*κ*) and quadratic-weighted kappa score^21^ (*κ*_quad_) were used, along with the grade group prediction accuracy (accuracy).

In the mechanism analysis of the proposed system, the features extracted via the trained cancer detection model were shown to be distinguishable according to the Gleason pattern. Furthermore, in the grade group prediction, the system worked presumably following the method of assessing the relative ratio of Gleason patterns by pathologists.

## Result

### Performance analysis

A total of 788 cases (7600 WSIs) from two institutions HUMC and KUGH were used for this study after the quality check. It is notable that each case corresponds to a different patient. In the holistic experimental setting, 99 cases (936 WSIs) randomly selected from the entire dataset per category per institution were used for the internal validation, and the remaining 689 cases (6664 WSIs) were used for the discovery, as detailed in Table 1. More specifically, the discovery WSIs were split into 5716 WSIs for training and 948 for tuning. Among the training WSIs, randomly chosen 5206 WSIs were used for training in the additional experiment. In the inter-institutional setting, 621 cases (6071 WSIs) from HUMC were used for the discovery, and 167 cases (1529 WSIs) from the KUGH were used for the internal validation. For the external validation, 244 tissue microarray (TMA) images of the Gleason 2019 Challenge^18,19^ training dataset were evaluated with the model trained in the holistic setting.

**Table 1:** Number of slides and cases in the entire dataset for this study.

Category	Discovery (HUMC)	Discovery (KUGH)	Validation (HUMC)	Validation (KUGH)	Gleason2019
Benign	3537 (490)	604 (109)	439 (64)	89 (15)	26
	66.3%	45.4%	59.6%	44.5%	10.7%
Grade group 1	570 (311)	134 (67)	76 (42)	19 (10)	70
(Gleason score ≤ 6)	10.7%	10.1%	10.3%	9.5%	28.7%
Grade group 2	379 (190)	80 (55)	68 (33)	14 (9)	23
(Gleason score 7 = 3 + 4)	7.1%	6.0%	9.2%	7.0%	9.4%
Grade group 3	274 (142)	231 (87)	51 (23)	40 (12)	21
(Gleason score 7 = 4 + 3)	5.1%	17.4%	6.9%	20.0%	8.6%
Grade group 4	300 (132)	118 (57)	47 (18)	20 (10)	101
(Gleason score 8)	5.6%	8.9%	6.4%	10.0%	41.4%
Grade group 5	275 (90)	162 (43)	55 (14)	18 (6)	3
(Gleason score ≥ 9)	5.2%	12.2%	7.5%	9.0%	1.2%
Total	5,335 (543)	1,329 (146)	736 (78)	200 (21)	244
	100%	100%	100%	100%	100%

The ratio of each category is also presented per each dataset type and institution. As each case can contain multiple slides from different categories, the sum of the cases for each category does not coincide with the total cases. For Gleason 2019, the case id couldn’t be identified for each TMA image.

**Fig. 1:**
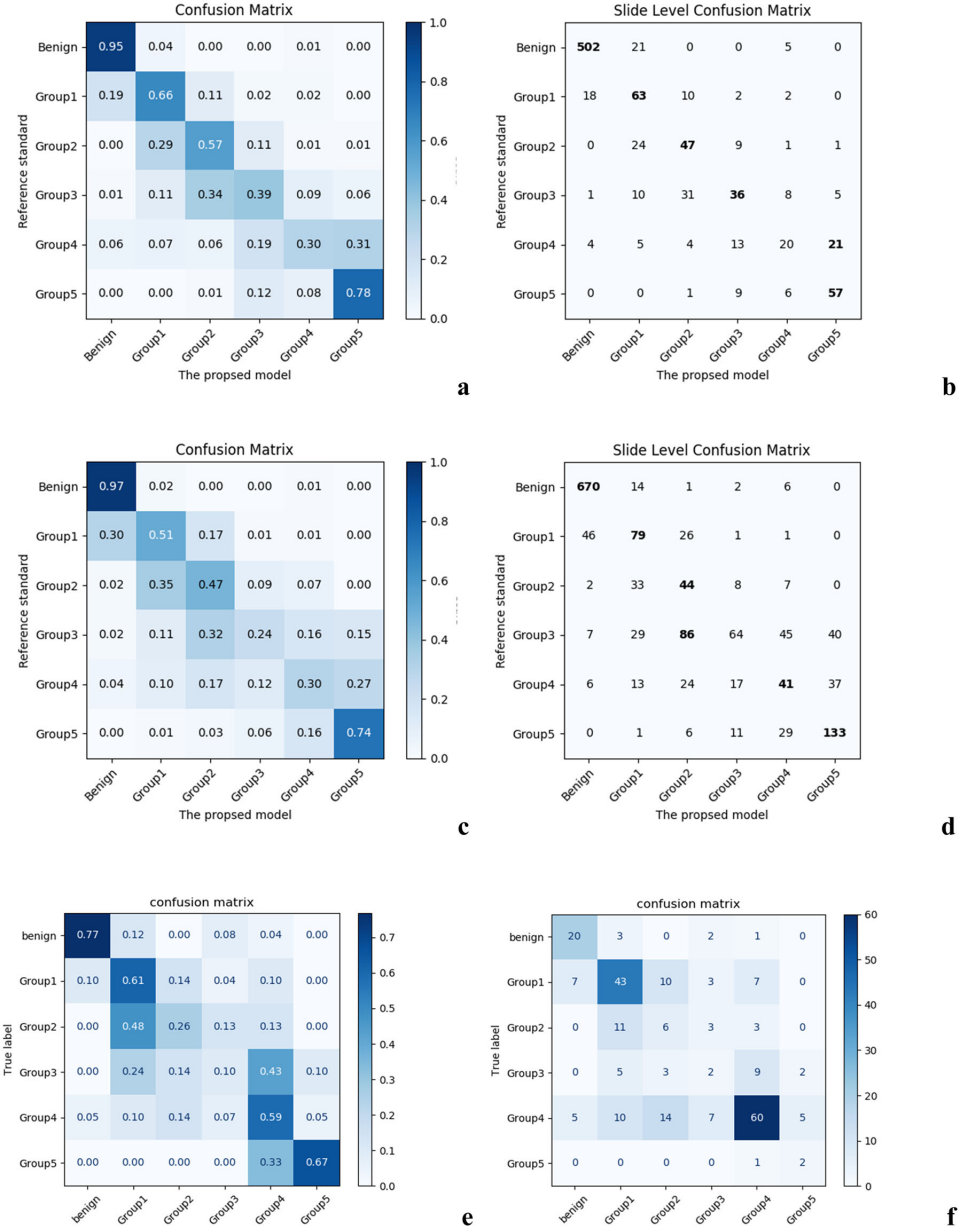
Slide-level confusion matrices between the proposed model and the reference standard in grade group prediction in the holistic setting. **a** normalized, **b** original; in the inter-institutional setting: **c** normalized, **d** original; in the external validation setting: **e** normalized, **f** original.

**Fig. 2:**
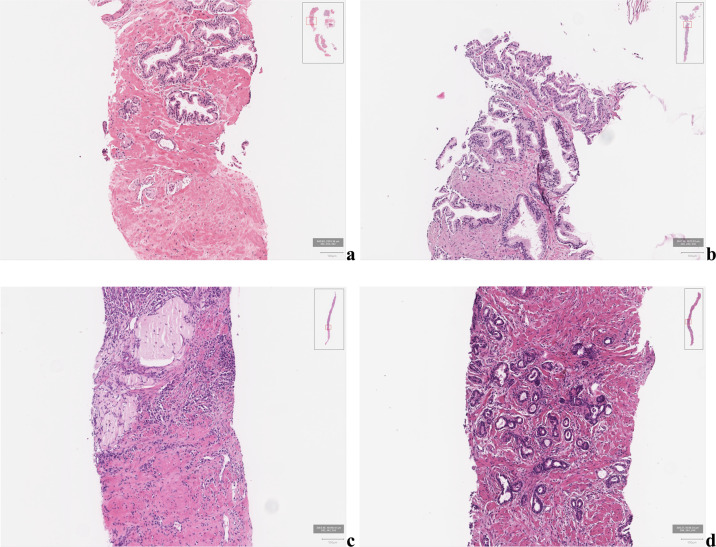
Sample patch images from failure cases (Hematoxylin-eosin stain, ×200). False-negative cases showed small-sized cancers, which consisted of only several cancer glands, or cancer glands located on the outer sample margin of the WSI. **a** was diagnosed as grade group 1 and **b** as grade group 4 in the reference standard, respectively. False-positive cases often exhibited diffuse infiltration of lymphocytes and atrophic glands. The prediction of the model was grade group 4 for (**c**) and grade group 1 (**d**), respectively.

In the holistic setting, for the WSI-level cancer detection task, the first stage model exhibited the receiver operating characteristic (ROC) area under the curve (AUC) value of 0.983 (95% CI: 0.964–1.000) and the precision-recall (PR) AUC value of 0.984 (95% CI: 0.965–1.000) on the validation dataset. The cancer detection accuracy, sensitivity, and specificity were 94.7% (95% CI: 91.4–98.0%), 0.936 (95% CI: 0.900–0.972), and 0.960 (95% CI: 0.931–0.989), respectively. For the grade group prediction, the second stage model showed the accuracy of 77.5% (95% CI: 72.3–82.7%), *κ* of 0.650 (95% CI: 0.570–0.730), and *κ*_quad_ of 0.897 (95% CI: 0.815–0.979). The confusion matrices are depicted in Fig. 1 (a, b). Representative patch images sampled from the model failure case (false positive and negative) WSIs are presented in Fig. 2. Representative patch images sampled from the WSIs in each predicted category are shown in Supplementary Fig. 1. In the additional experiment where the second stage model was trained on the subset of data, the model showed the accuracy of 69.3%, *κ* of 0.521, and *κ*_quad_ of 0.824.

In the inter-institutional setting, as a WSI-level cancer detector, the first stage model exhibited the ROC AUC value of 0.982 (95% CI: 0.967–0.997) and the PR AUC value of 0.984 (95% CI: 0.970–0.998). For the second stage model, the accuracy dropped to 67.4% (95% CI: 63.2–71.6%), *κ* to 0.553 (95% CI: 0.495–0.610), and *κ*_quad_ to 0.880 (95% CI: 0.822–0.938). The confusion matrices are presented in Fig. 1 (c, d).

In the external validation using Gleason 2019 dataset, the first stage model exhibited the ROC AUC of 0.943 (95% CI: 0.913–0.973) and the PR AUC of 0.985 (95% CI: 0.972–0.998). For the second stage model, the accuracy was 54.5% (95% CI: 48.3–60.8%), *κ* was 0.389 (95% CI: 0.305–0.473), and *κ*_quad_ was 0.634 (95% CI: 0.468–0.800).

### Comparative analysis

The performance comparison result of the proposed model to three baseline methods is presented in Table 2. More specifically, the grade group prediction accuracy dropped from 77.5% of the proposed model to 72.6% (95% CI: 67.2–78.1%) when an ImageNet pre-trained model was used for the first stage, to 75.6% (95% CI: 70.3–80.9%) when a multi-class MIL method^22^ was used for training the first-stage model, and to 67.3% (95% CI: 61.6–73.0%) when the CLAM^23^ was used.

**Table 2:** Performance indices of the proposed system and baseline methods.

Value (95% CI)	The proposed system	Using ImageNet pre-trained model	Using multi-class MIL-trained model	CLAM
Accuracy (%)	**77.5** (72.3–82.7)	72.6 (67.2–78.1)	*75.6* (70.3–80.9)	67.3 (61.6–73.0)
*κ*	**0.650** (0.570–0.730)	0.559 (0.471–0.647)	*0.622* (0.540–0.703)	0.469 (0.376–0.562)
*κ*_quad_	*0.897* (0.815–0.979)	0.845 (0.746–0.945)	**0.901** (0.819–0.982)	0.779 (0.658–0.900)

For each criteria, the maximum value is set bold-faced, and the second maximum is set italic.

### Mechanism evaluation

**Fig. 3:**
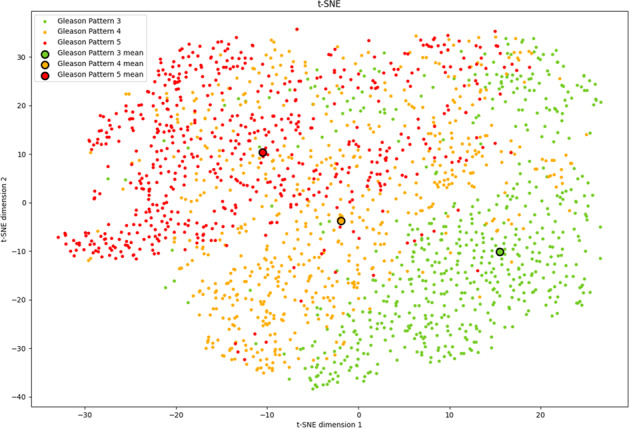
t-SNE data visualization of the feature vectors of the Gleason pattern 3/4/5 image patches embedded by the first stage model for perplexity 50 and 1000 iterations. Bigger dots correspond to the mean feature vectors.

We analyzed the output feature vectors of the first stage model through the t-distributed stochastic neighbor embedding (t-SNE) data visualization to evaluate the overlaps among Gleason pattern-wise feature distributions (Fig. 3). The diagrams of t-SNE plots for three different perplexity values are shown in Supplementary Fig. 2. In this figure, each dot corresponds to a feature vector, and the dots of the same color correspond to the feature vectors of the same label, indicating that the vectors from the patch images of the same Gleason pattern. Bigger dots correspond to the mean feature vectors of each label. It shows that the first stage model embeds different patterns to the different positions in feature space, despite the fact that the model only learned for the presence or absence of cancer.

Table 3 shows the mean and deviation values of the actual output of the second stage model for a set of combination ratios between Gleason patterns 3 and 4, evaluated on 30 synthetic WSIs assembled from 5 pairs of WSIs in the validation set chosen to have similar amounts of Gleason patterns 3 and 4, respectively. The graphs showing the model outputs according to the combination ratio for each pair are presented in Supplementary Fig. 3.

**Table 3:** Mean and deviation values of the actual output of the second stage model for a set of combination ratios between Gleason patterns 3 and 4.

GP3: GP4	100%: 0%	80%: 20%	60%: 40%	40%: 60%	20%: 80%	0%: 100%
benign	0.00 ± 0.00	0.00 ± 0.00	0.00 ± 0.00	0.00 ± 0.00	0.00 ± 0.00	0.01 ± 0.00
grade group 1	0.30 ± 0.20^c,2^	0.05 ± 0.10	0.02 ± 0.02	0.01 ± 0.03	0.01 ± 0.01	0.00 ± 0.01
grade group 2	0.54 ± 0.13^1^	0.55 ± 0.08^c,1^	0.37 ± 0.21^c,2^	0.28 ± 0.26^2^	0.20 ± 0.15	0.09 ± 0.14
grade group 3	0.14 ± 0.08	0.33 ± 0.13^2^	0.44 ± 0.07^1^	0.44 ± 0.10^c,1^	0.40 ± 0.10^c,1^	0.28 ± 0.15
grade group 4	0.02 ± 0.01	0.06 ± 0.05	0.14 ± 0.12	0.19 ± 0.13	0.25 ± 0.13^2^	0.33 ± 0.09^c,1^
grade group 5	0.00 ± 0.00	0.01 ± 0.01	0.04 ± 0.07	0.07 ± 0.08	0.11 ± 0.14	0.29 ± 0.21^2^

For each combination ratio, superscript c indicate the accurate category, while the category with the maximum mean value is with superscript 1, and the second maximum is with superscript 2.

## Discussion

We proposed YAAGGS, a novel two-stage WSI prostate cancer grade group prediction system trained only with slide labels. In the holistic setting, the proposed system yielded the kappa (*κ*) value of 0.650 compared to the pathologist-based reference standards. In literature, the inter-observer Gleason scoring concordance rates measured in *κ* vary in the range of 0.40–0.50 between general pathologists, and 0.56–0.70 for urologic pathologists.^6,7,24,25^

Certain cases of grade groups 3 and 4 were predicted as benign. By reviewing such cases, we found that the model missed small-sized cancer which consisted of only several cancer glands, or cancer glands located on the outer sample margin of the WSI (Fig. 2a, b). False-positive cases often exhibited diffuse infiltration of lymphocytes and atrophic glands (Fig. 2c, d).

In the inter-institutional setting, the system yielded a lower but still decent grading performance (*κ* = 0.553). To analyze the cause of this performance degradation, we performed an additional experiment in the holistic setting with the same number of training slides as in the inter-institutional setting (*n* = 5206) and obtained a similar performance (*κ* = 0.521), which supports the assumption that this performance degradation is at least partly due to the size reduction of the training dataset.

The prediction performance of the model was degraded when externally validated using the Gleason 2019 dataset. The difference in color distribution can be considered as the cause of the performance decline. The stain color distribution of the Gleason 2019 dataset was visually different from the HUMC and KUGH datasets. We have incorporated color augmentation in model training to cope with the stain color variations among institutions, but it might not have been sufficient to deal with the external dataset.

There exist several studies that propose deep learning models for prostate cancer grading that are trained without region-level manual annotations. Bulten et al. reported a deep learning model trained with the semi-automatic region-level annotation technique and slide-level annotations to show *κ*_quad_ of 0.918 (95% CI: 0.891–0.941).^17^ Similarly, Ström et al. developed an ensemble of deep learning models trained with the automatically generated region-level annotations from pen marks and slide-level annotations, yielding the linear-weighted kappa score (*κ*_lin_) of 0.83.^16^ The study by Li et al. likewise introduced a WSI classification model trained by a weakly supervised learning method.^26^ However, unlike our proposed system that requires no side-used algorithms to create or enhance the data, the first two studies relied on explicit complex algorithms to create annotations of sufficient quality. The study by Li et al. differs from ours in that its second stage model requires only cancer patch images to be fed.

In the comparative analysis, our proposed method outperformed other baseline methods. Using an ImageNet pre-trained model as a feature extractor is a common technique to overcome the lack of data.^27^ However, we discovered that incorporating a model that can extract prostate cancer-specific histological features into our proposed system leads to better performance. We also discovered that there was no vivid performance change when using a feature extractor that optimized for the Gleason pattern discrimination instead of the original one that just for cancer detection. This supports the assumption that our first stage model might have actually learned the Gleason pattern-specific features.

While the attention-based MIL method is known as a powerful one in the weakly supervised learning setting^23,28,29^, adopting the CLAM^23^ model was not effective in our case even with some extent of hyperparameter tuning. It would be valuable to discover further the reason for this ineffectiveness, but it seems outside the scope of this study.

In addition to the statistical analysis of the prediction performance of the proposed model, we attempted to analyze the model mechanism in terms of its analogy with the diagnosis process of the pathologist. Diagnosing the grade group involves two types of tasks: recognition of Gleason patterns and estimation of respective portions. Therefore, we performed the mechanism analysis in two steps. In the first step, we evaluated whether the model distinguishes the Gleason patterns. The second step assessed the proportion sensitivity of the model, which is necessary to grade prostate cancer accurately. Figure 3 represents the feature vector distributions for the patch images containing Gleason patterns 3, 4, or 5 and shows that apparent differences exist among Gleason pattern-wise distributions. Thus, we assume that the first stage model assigns distinguishable features to different Gleason patterns, enabling the second stage model to predict the grade groups based on the generated feature map.

Next, we analyzed the change in the actual output of the second stage model according to the combination ratio of Gleason patterns to evaluate the proportion sensitivity. As shown in Table 3, the model reacts sensitively as the proportion of Gleason pattern 4 increases, such that the value for grade group 1 continually decreases, both the values for grade groups 4 and 5 increase, and both the values for grade groups 2 and 3 increase to a certain point and then decreases. Thus, we found that the second stage model is sensitive to the proportion of Gleason patterns. Notably, we could perform a similar analysis with Gleason patterns 3 and 5; however, we did not conduct the analysis because a slight amount of Gleason pattern 5 results in the grade group 4 or higher, making the analysis less sensitive. Even a very small amount of Gleason pattern 5, as long as it is observed, is reflected in the Gleason score because it is the highest pattern, so the Gleason score does not change significantly by subtle differences in the amount of Gleason pattern 5. On the other hand, a Gleason pattern score lower than the most common score is ignored unless it exceeds 5% of the total cancer area. In practice, the pathologist assesses the glass slide at low magnification (×4 and ×10 objectives) when using a light microscope as an initial diagnostic step. Then by increasing magnification, they determine the detailed ratio of each Gleason pattern and make a final diagnosis. Considering the results, we thought that our second stage model operates in the manner reflecting quantitative change as the pathologist does.

Nevertheless, there are several limitations related to the size and quality of the study data. First, the reference standard was not strongly established. Gleason grading is known to be highly variable among pathologists. Grading among experts is more reproducible than among general pathologists. However, our results were evaluated using a weak reference standard derived from either a single pathologist or the original hospital diagnosis. While this study aimed to lower the development cost of the artificial intelligence systems, the clinical utility of the study would be better proven with more strongly established reference standards. Second, the inter-institutional generalization power is not well demonstrated due to the limited case volume.

Additionally, as our study data is from two institutions, one graded with either the 2005 or the 2014 ISUP Gleason grading guidelines and the other graded with the 2014 guidelines. For HUMC data, the data collection period was very long (9 years) and we were unable to analyze in detail whether the guideline changes and inter-observer variability had an effect on the validation tests. Future research is necessary through the participation of an increased number of hospitals and pathologists.

In summary, we presented a novel weakly supervised deep learning-based automated Gleason grading system trained only from slide-level annotations using the MIL method. We expect that this study can possibly contribute to the development of AI systems to diagnose different types of cancer, which have several morphological grades without constructing region-level annotation.

## Methods

### Data

Hematoxylin and eosin (H&E) stained glass slides each containing a single prostate needle biopsy core and their respective diagnoses were collected from two hospitals: HUMC, Seoul, Korea (Institutional Review Board Approval No. 2018-10-010-002) and KUGH, Seoul, Korea (Institutional Review Board Approval No. K2017-4488-001). Prostatic needle biopsies were performed from 2009 to 2017 and from 2010 to 2016 at HUMC and KUGH, respectively. The Institutional Review Boards of the two hospitals approved this retrospective study and waived the requirement for informed consent. We confirm that all experiments were performed according to relevant guidelines and regulations.

The slides were digitized using Aperio AT2 scanners (Leica Biosystems Inc., Vista, CA, USA), at ×40 magnification, (i.e., resolution of 0.25 µm/pixel). After digitization, a pathologist, H.C. blinded to the pathologic diagnosis performed a manual quality check. Exclusion criteria included:

1. slides for a tissue biopsied from organs other than the prostate or surrounding tissues,
2. immunohistochemistry or special stains slides,
3. slides with inadequate quality for pathologic diagnosis, including severe out-of-focusing or indelible markings.

The Gleason scores from the original hospital diagnoses were converted into the corresponding grade groups and used as the slide label annotations and the reference standard for the HUMC dataset. During the period from 2009 to 2017, five surgical pathologists, whose experience ranged from 1 to 20 years, worked at the HUMC on average, and one of them was a genitourinary pathologist. For the KUGH dataset, a Korea board-certified pathologist with 9 years of experience reviewed all WSIs according to the 2014 ISUP Gleason grading guidelines and created the slide label annotations and the reference standard. For the external validation, the Gleason 2019 Challenge data was used.^18,19^ These patients had undergone radical prostatectomy at the Vancouver General Hospital between June 1997 and June 2011. The TMAs were prepared in the same lab and with the same procedures at the Vancouver Prostate Centre in Vancouver, Canada. The TMAs had been stained in H&E and scanned at 40x magnification with a SCN400 Slide Scanner (Leica Microsystems, Wetzlar, Germany). Six pathologists were asked to annotate the TMA images in detail. The pathologists had 27, 15, 1, 24, 17, and 5 years of experience. Four of the pathologists annotated all 333 cores. The other two pathologists annotated 191 and 92 of the cores. Pixel-wise majority voting is used to build the “ground truth label”.^18^ The ground-truth Gleason grade group for each TMA image is derived by simple pixel counting algorithm according to the 2014 ISUP Gleason grading guidelines for needle biopsy^30^. But we ignored any pattern that is occupying <1% of the total area, to deal with the noise induced by the pixel-level majority vote process. We validated our model only on 244 training images because the ground truth labels were not available for the remaining test images.

### Two-stage WSI classification model

The proposed YAAGGS classifies the input WSIs into Gleason grade groups in two stages. In the first stage of feature extraction, patch images of size 360 × 360 pixels covering the entire slide area are extracted from the input WSI at 10× magnification and fed into the first stage CNN model to extract 1024-dimensional feature vectors. The extracted feature vectors were aligned according to the locations of corresponding patch images to be assembled into a 1024-channel two-dimensional feature map. The second stage CNN model accepts the feature maps as input and classifies them into one of six categories: benign, grade group 1, grade group 2, grade group 3, grade group 4, and grade group 5.

### Model development

Throughout the study, WSIs in the discovery set were randomly distributed into the training and tuning sets with the ratio of 6:1. Both the first stage and second stage models were trained on the same training set, and the performances of the models were evaluated on the same tuning set per each training epoch to choose the best performing model parameters.

The first stage model was trained to classify input patch images into two classes, benign and cancer. The MIL method was used to train the first-stage model, as in another study.^31^ We adopted DenseNet-121 as our first stage model architecture and used the ImageNet pre-trained weight parameters as the initial model parameter values.^32^ The condition of the first stage model training is as follows: Initial learning rate was 0.01. The stochastic gradient descent (SGD) optimizer with 0.9 momentum and 1e-5 weight decay was used. The learning rate was scheduled to be multiplied by 0.1 per every 25 epoch. The total training epoch was 100 epochs and the mini-batch size was 128. Applied data augmentations were random horizontal flip, random 90° rotation, and random change of the brightness, contrast, saturation, and hue as amounts of ±0.1, ±0.3, ±0.3, and ±0.05, respectively. The model with the best PR AUC score at tuning set was chosen. After the training step, the last hidden layer output of the model was tapped to obtain 1024-dimensional feature vectors for the input patch images.

To train the second-stage model, we converted WSIs in the discovery set into feature maps, as described above, using the first-stage model. More specifically, patch images of 360 × 360 pixel size in three channels were converted into images of 1 × 1 pixel size in 1024 channels, resulting in the width and height of each WSI resized by 1/360. Accordingly, WSIs equal to or less than 2 × 2 cm^2^ can be safely converted into 1024-channel 64 × 64 pixel-sized feature maps. The second stage model was trained to classify these feature maps into grade groups, based on the converted discovery set.

**Fig. 4:**
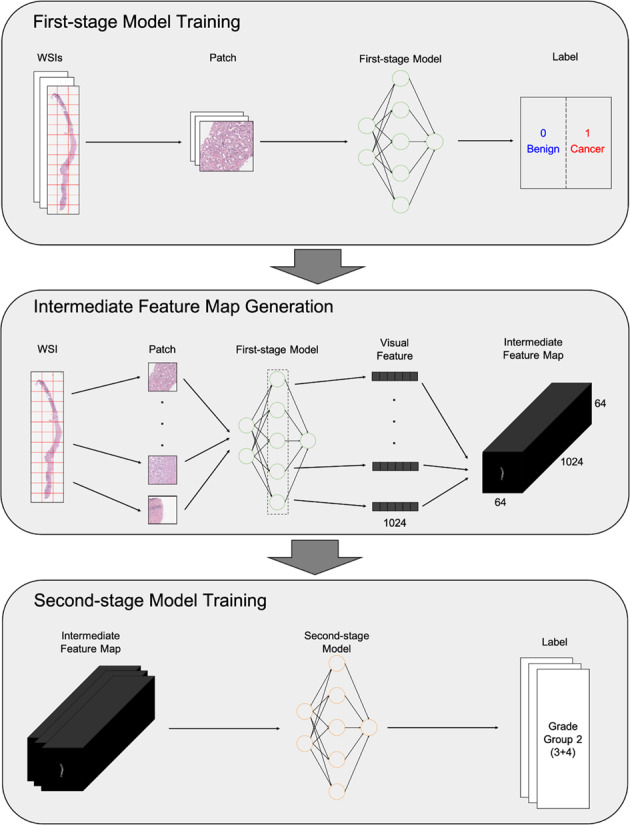
Model training process of YAAGGS. The model classifies the input WSIs into Gleason grade groups in two stages. In the first stage of feature extraction, patch images of size 360 × 360 pixels covering the entire slide area are extracted from the input WSI at 10× magnification and fed into the first stage CNN model to extract 1024-dimensional feature vectors. The extracted feature vectors were aligned according to the locations of corresponding patch images to be assembled into a 1024-channel two-dimensional feature map. The second stage CNN model accepts the feature maps as input and classifies them into one of six categories: benign, grade group 1, grade group 2, grade group 3, grade group 4, and grade group 5.

The architecture of the second stage model consists of two parts. The front part is composed of five layers of a 1 × 1 kernel convolution with batch-normalization and rectified linear unit (ReLU) non-linear activation, and the second part is composed of 16 blocks of convolution layer with the residual connection.^33^ The exact configuration of the second-stage model and the residual block is presented in Supplementary Table 1 and Supplementary Table 2, respectively. In training the second-stage model, we minimize the weighted cross-entropy loss as the objective function, as a class imbalance exists in the discovery dataset. The weight parameter was 1, 1, 1.5, 1.4, 1.7, and 1.6 for each class from ‘benign’ to “grade group 5”. The training condition of the second stage model was as follows. The initial learning rate was 0.1. The SGD optimizer was used with 0.9 momentum and 1e-5 weight decay. The learning rate scheduler used was a step learning rate scheduler with 40 epoch step size and 0.1 decay rate. The total training epoch was 150 epochs and mini batch size was 256. Applied data augmentations were random shift with –5 to 5 pixel range horizontally and vertically, random vertical flip, and random 90° rotation. The model with the best *κ*_quad_ score at the tuning set was selected. Figure 4 depicts the entire training process.

### Performance analysis

In the holistic setting, we trained our model using a part of HUMC + KUGH (6664 slides) and validated it for the remainder (936 slides). To analyze the generalization power of the model across the institutional boundary, we trained our model using HUMC and validated its performance on KUGH in the inter-institutional setting. An additional experiment was conducted in the holistic setting, using randomly sampled 5,206 training slides (uniformly chosen from 5716 slides) while tuning and validation slides were fixed, to analyze the effect of the size of the training data on the model performance.

For the external validation, we validated the model trained in the holistic setting, using publicly available data from the Gleason 2019 challenge.^18,19^

### Comparative analysis

To evaluate the impact of the proposed method, we compared its performance with several baseline methods. The first method uses a pre-trained model as a feature extractor instead of the proposed first-stage model. We used an ImageNet pre-trained DenseNet-121 model here with no further training. The second stage model is then trained based on the feature vector output of the pre-trained model, extracted as proposed. The second method adopts a multi-class MIL method to train the first-stage model. We modified the DenseNet-121 model to have four binary classification outputs, each for benign, Gleason patterns 3, 4, and 5 and trained it with the method proposed by Pathak et al.^22^ The second stage model is then trained as before. The last approach is based on a recently proposed weakly supervised learning method named CLAM.^23^ We used the authors’ code to train the CLAM model to classify WSIs into one of six categories, as the proposed second stage model does. Because the optimal settings may vary for different tasks, we searched for optimal hyperparameters within a reasonable range; Patch-wise feature vectors were extracted via an ImageNet pre-trained ResNet-50 and DenseNet-121 model and aggregated by attention-based pooling. And the feature vector was classified by linear layers of size [128, 256, 512, 1024]. We tried bag weights of [0.0, 0.3, 0.5, 0.7, 1.0]. We used Stochastic Gradient Descent (SGD) optimizer with momentum 0.9 and weight decay 10^−4^, with a learning rate between 10^−1^ and 10^−5^. Early stopping is used based on the best validation quadratic kappa score.

### Mechanism evaluation

To evaluate whether the first stage model distinguishes the Gleason patterns, we attempted to visualize the feature vectors extracted by the first stage model onto a two-dimensional space using the t-SNE dimensionality reduction.^34^ We randomly sampled 600 cancer image patches from the WSIs in the validation dataset with the Gleason score 3 + 3, 4 + 4, or 5 + 5 each. In all, 1800 image patches were embedded into 1024-dimensional space by the first stage model and processed through the t-SNE technique, and the result was visualized as a two-dimensional plot. While the learning rate of the t-SNE algorithm was fixed to 200, the number of iterations fixed to 1000, and the perplexity hyperparameter varied from 5 to 1,000. We got consistent results regardless of the parameters. The visualization in Fig. 3 is the results of perplexity 50 and 1000 iterations.

To assess the proportion sensitivity of the second-stage model, we performed an experiment to measure the change in the grade group probabilities according to the proportions of the Gleason patterns. The experiment was performed as follows. First, we sampled five non-overlapping pairs of WSIs, each with Gleason score 3 + 3 and 4 + 4, from the validation dataset. Next, we synthesized virtual WSIs according to the six predefined combination ratio values, namely “‘100%: 0%”, “80%: 20%”, “60%: 40%”, “40%: 60%”, “20%: 80%”, and “0%: 100%”, from each sampled WSI pair. For example, a synthetic WSI with “60%: 40%” combination ratio is a horizontal concatenation of 60% of 3 + 3 WSI and 40% of 4 + 4 WSI. Subsequently, the synthesized WSIs were fed into the first stage model to generate feature maps, which were then processed by the second stage model to generate output values. Supplementary Fig. 4 depicts the overall workflow of this experiment.

### Statistical evaluation

As we trained the first stage model to identify cancer-specific visual features, we evaluated its performance as a cancer detector. The output of the model for a given patch image is its predicted probability of containing a cancer lesion. We applied max pooling to the model outputs to obtain the WSI-level probability of containing a cancer lesion. We measured both the ROC AUC and the PR AUC for the validation dataset. We also measured the sensitivity, specificity, and accuracy of the model with the threshold yielding the best f1-score.

To assess the performance of the second stage model as a grade group predictor, Cohen’s kappa score was measured between the model output and the reference standard on the validation dataset, both with and without quadratic weighting.^20,21^ The grade group prediction accuracy was also assessed. Confidence intervals for kappa statistics were computed based on the equation presented by McHugh.^35^ For other performance indices, such as accuracy, the normal approximation of the binomial confidence interval was used.^36^

### Reporting summary

Further information on research design is available in the Nature Research Reporting Summary linked to this article.

## Supplementary information

Supplementary Information

Reporting Summary

## Data Availability

The datasets used in this study are not publicly available at this moment due to data usage agreement restrictions. The de-identification process and usage of slides have been approved by the respective Institutional Review Boards (IRBs) of KUGH and HUMC. The datasets are available upon reasonable request and in order to use the data, qualified researchers should be approved by the institutional review boards of both institutions.

## Code availability

The source codes used in this study are copyrighted by Deep Bio Inc. and are available only under appropriate license agreement with the copyright holder.
