# Supplementary material for: Yet Another Automated Gleason Grading System (YAAGGS) by weakly supervised deep learning
Source: NPJ Digit Med. 2021 Jun 14;4:99. doi: 10.1038/s41746-021-00469-6 (PMC8203612; doi:10.1038/s41746-021-00469-6)
Supplement: Supplementary file 1 — Supplementary Information [file 41746_2021_469_MOESM1_ESM.pdf]

Supplementary Information

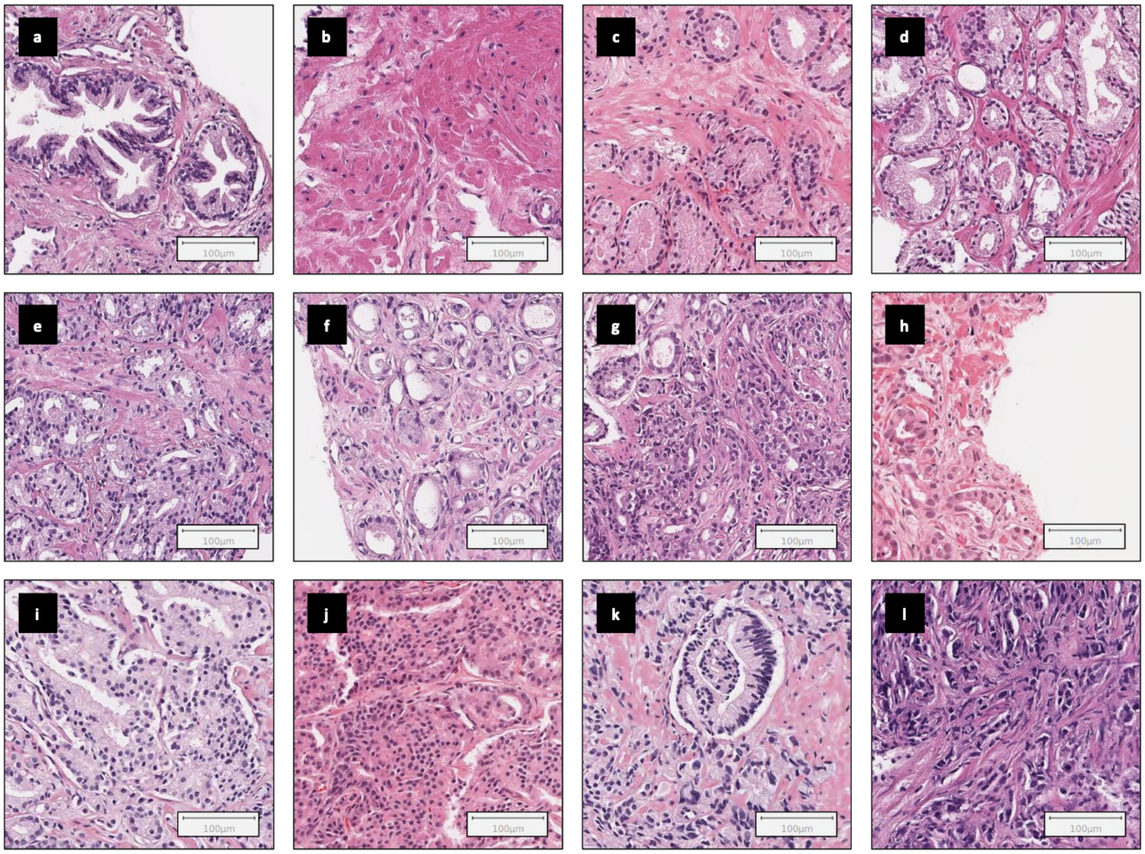

Supplementary Figure 1. Sample patch images from WSIs in validation set (360 × 360 pixels). After the WSIs were categorized according to the output of the proposed model, a number of patch images were randomly sampled from the WSIs for each category. A pathologist reviewed all sampled patch images to choose two of them representative for each category: (**a**, **b**) benign, (**c**, **d**) grade group 1, (**e**, **f**) grade group 2, (**g**, **h**) grade group 3, (**i**, **j**) grade group 4, and (**k**, **l**) grade group 5.

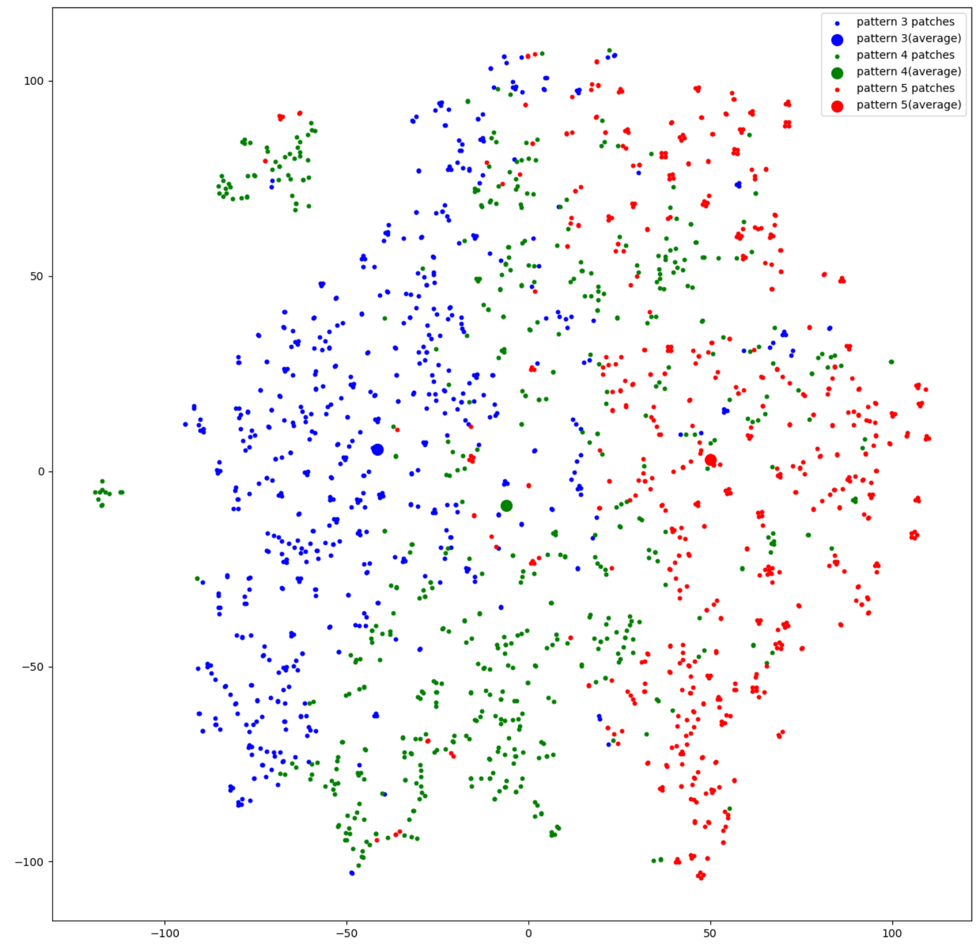

pattern 3 patches
pattern 3(average)
pattern 4 patches
pattern 4(average)
pattern 5 patches
pattern 5(average)
100
50
0
−50
−100
−100
−50
0
50
100

(a)

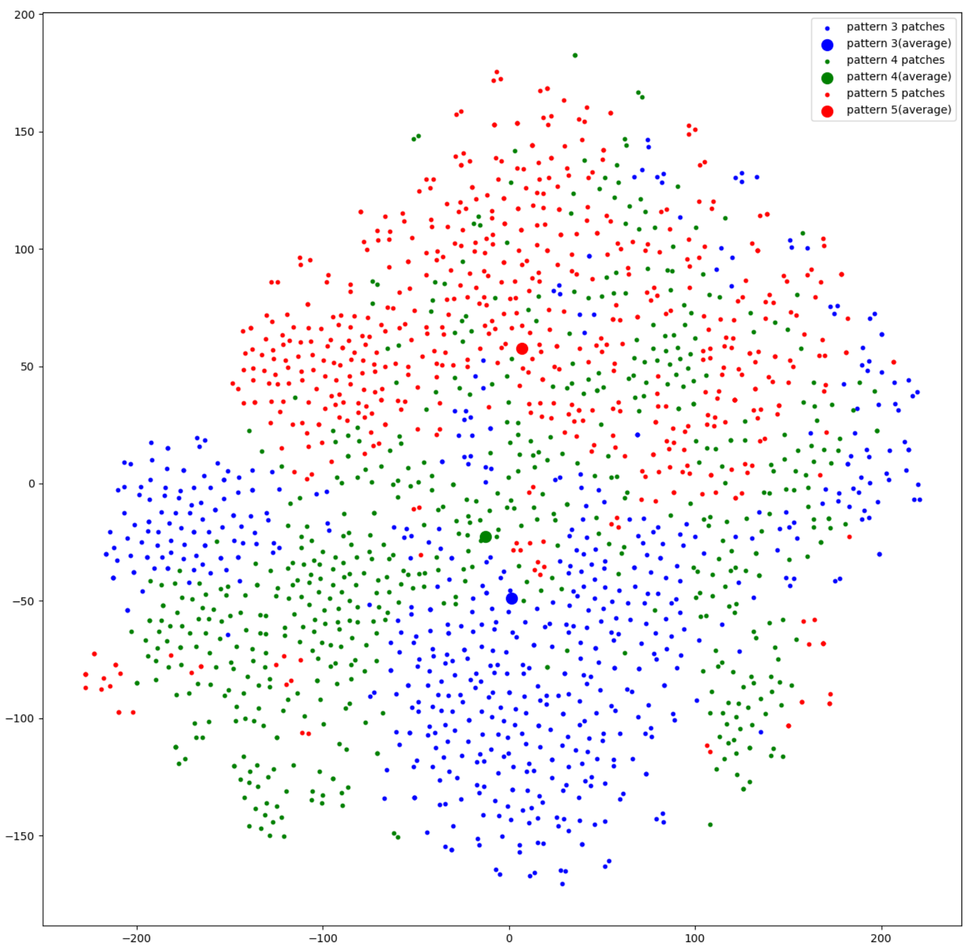
pattern 3 patches
pattern 3(average)
pattern 4 patches
pattern 4(average)
pattern 5 patches
pattern 5(average)
200
150
100
50
0
−50
−100
−150
−200
−100
0
100
200

(b)

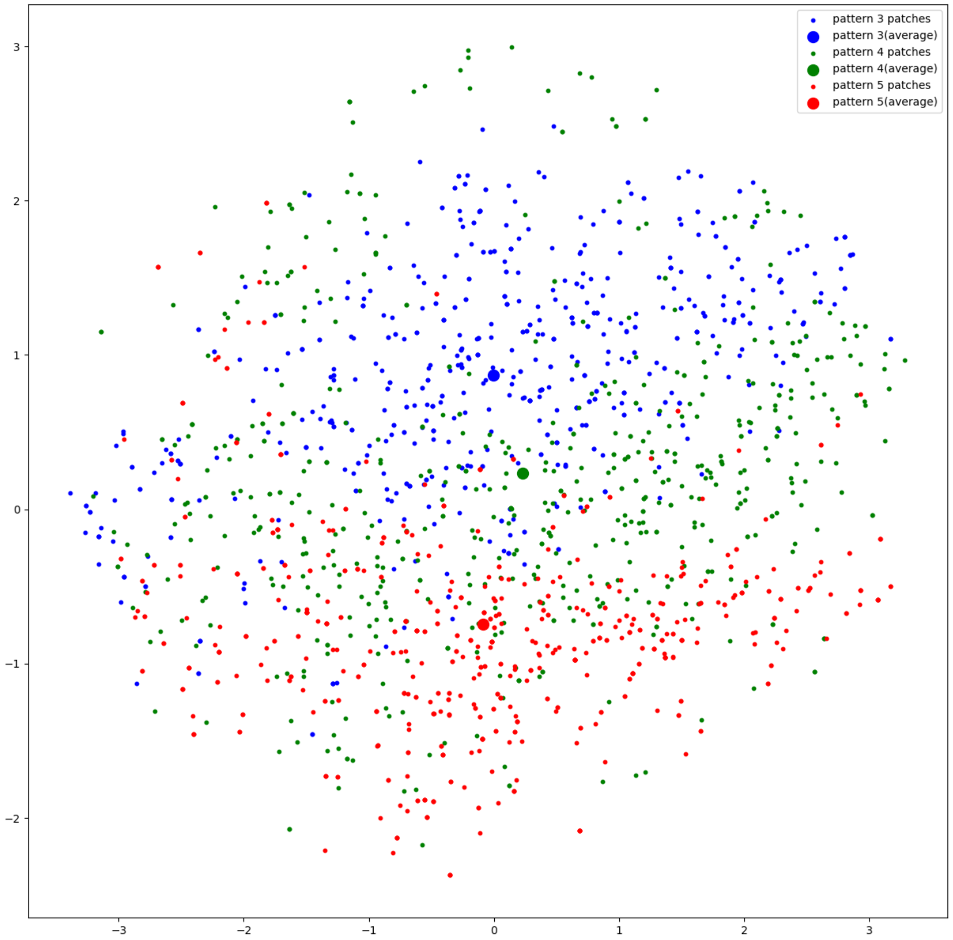

(c)

Supplementary Figure 2. t-SNE data visualization of the feature vectors of the Gleason pattern 3/4/5 image patches embedded by the first stage model, with 1,000 iterations and the perplexity parameter (**a**) 10, (**b**) 100, and (**c**) 1000. Bigger dots correspond to the mean feature vectors.

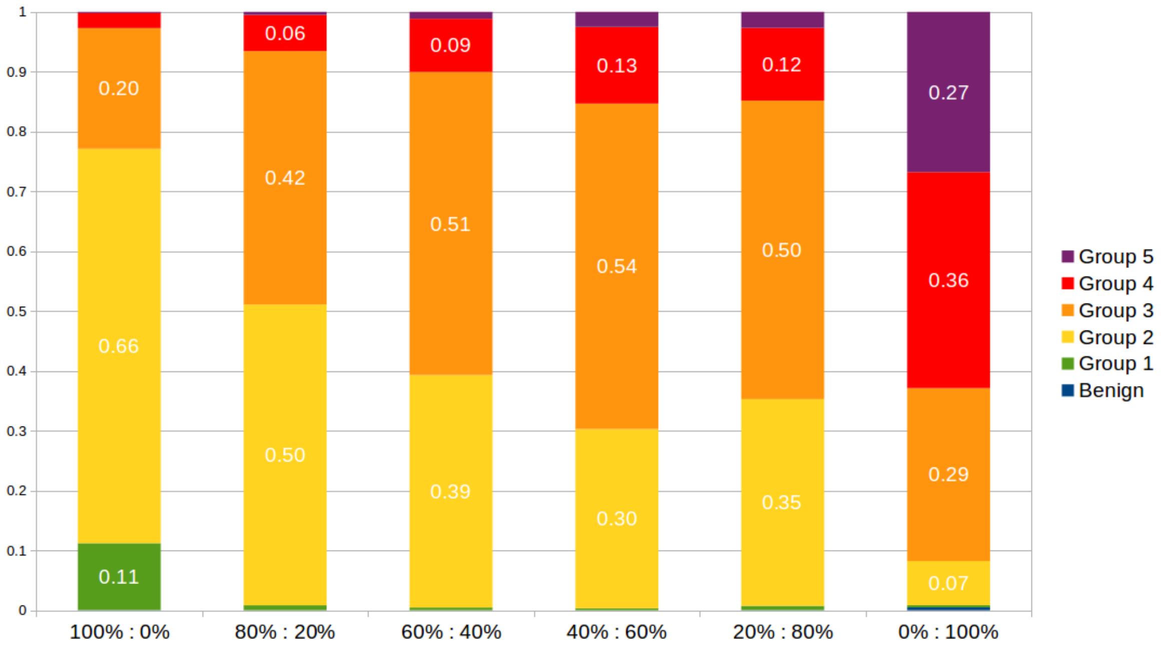

0.20
0.66
0.11
0.06
0.42
0.50
0.09
0.51
0.39
0.13
0.54
0.30
0.12
0.50
0.35
0.27
0.36
0.29
0.07
100% : 0%
80% : 20%
60% : 40%
40% : 60%
20% : 80%
0% : 100%
Group 5
Group 4
Group 3
Group 2
Group 1
Benign

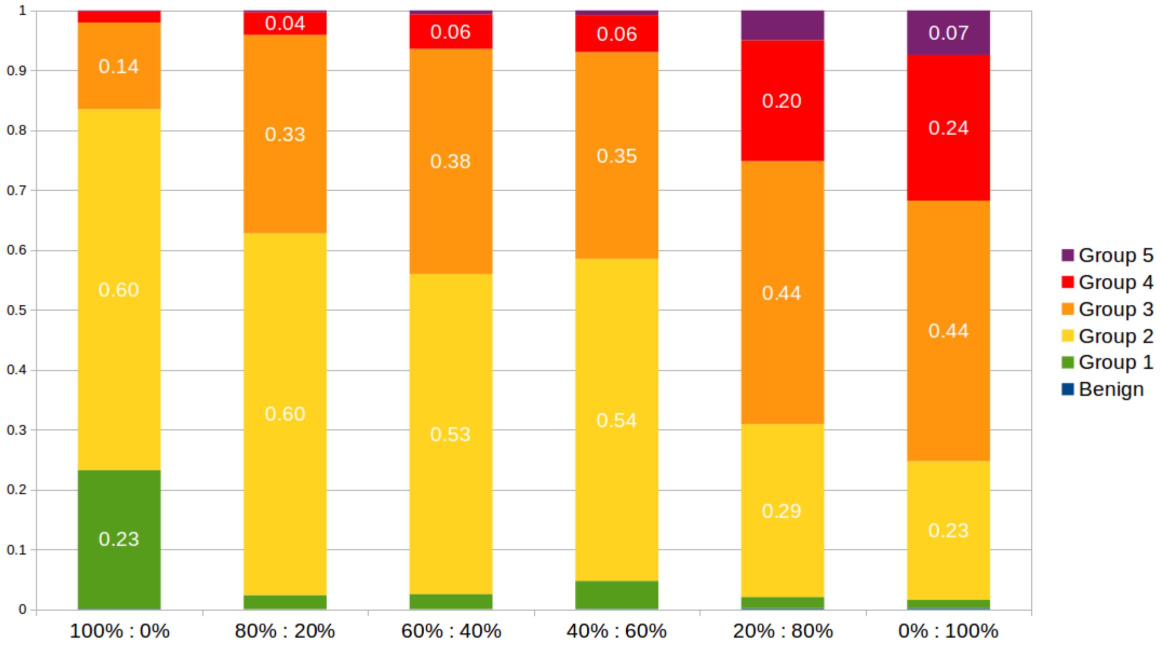

0.14
0.60
0.23
0.04
0.33
0.60
0.06
0.38
0.53
0.06
0.35
0.54
0.20
0.44
0.29
0.07
0.24
0.44
0.23
100% : 0%
80% : 20%
60% : 40%
40% : 60%
20% : 80%
0% : 100%
Group 5
Group 4
Group 3
Group 2
Group 1
Benign

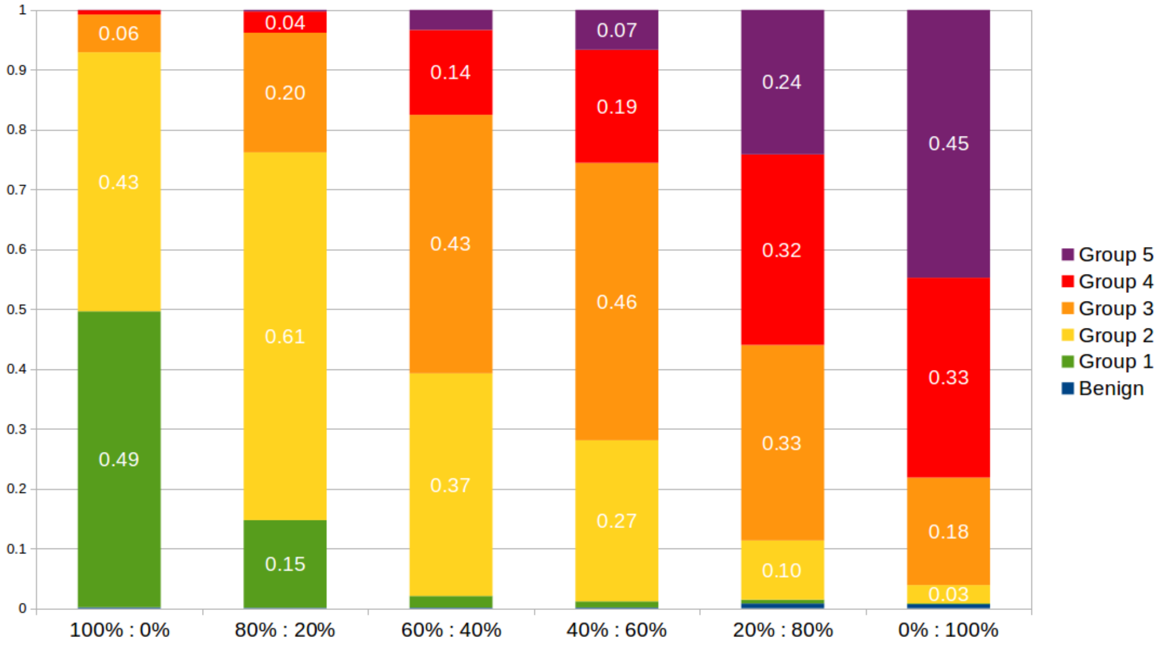

1
0.9
0.8
0.7
0.6
0.5
0.4
0.3
0.2
0.1
0
0.06
0.43
0.49
0.04
0.20
0.61
0.15
0.14
0.43
0.37
0.07
0.19
0.46
0.27
0.24
0.32
0.33
0.10
0.45
0.33
0.18
0.03
100% : 0%
80% : 20%
60% : 40%
40% : 60%
20% : 80%
0% : 100%
Group 5
Group 4
Group 3
Group 2
Group 1
Benign

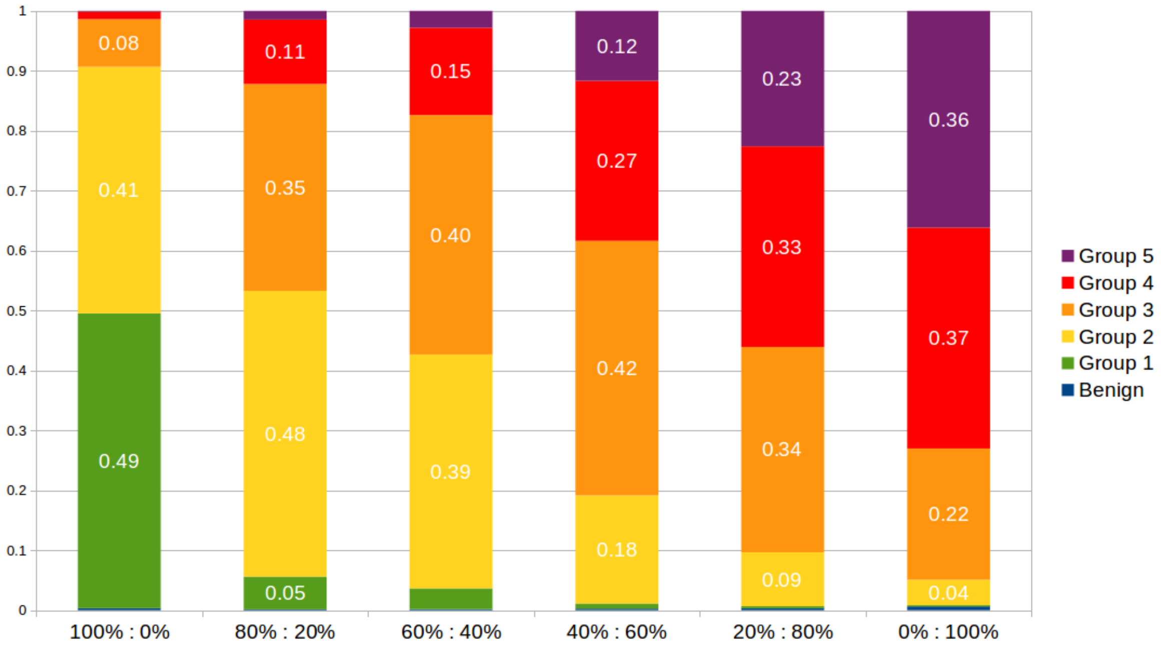

1
0.9
0.8
0.7
0.6
0.5
0.4
0.3
0.2
0.1
0
0.08
0.41
0.49
0.11
0.35
0.48
0.05
0.15
0.40
0.39
0.12
0.27
0.42
0.18
0.23
0.33
0.34
0.09
0.36
0.37
0.22
0.04
100% : 0%
80% : 20%
60% : 40%
40% : 60%
20% : 80%
0% : 100%
Group 5
Group 4
Group 3
Group 2
Group 1
Benign

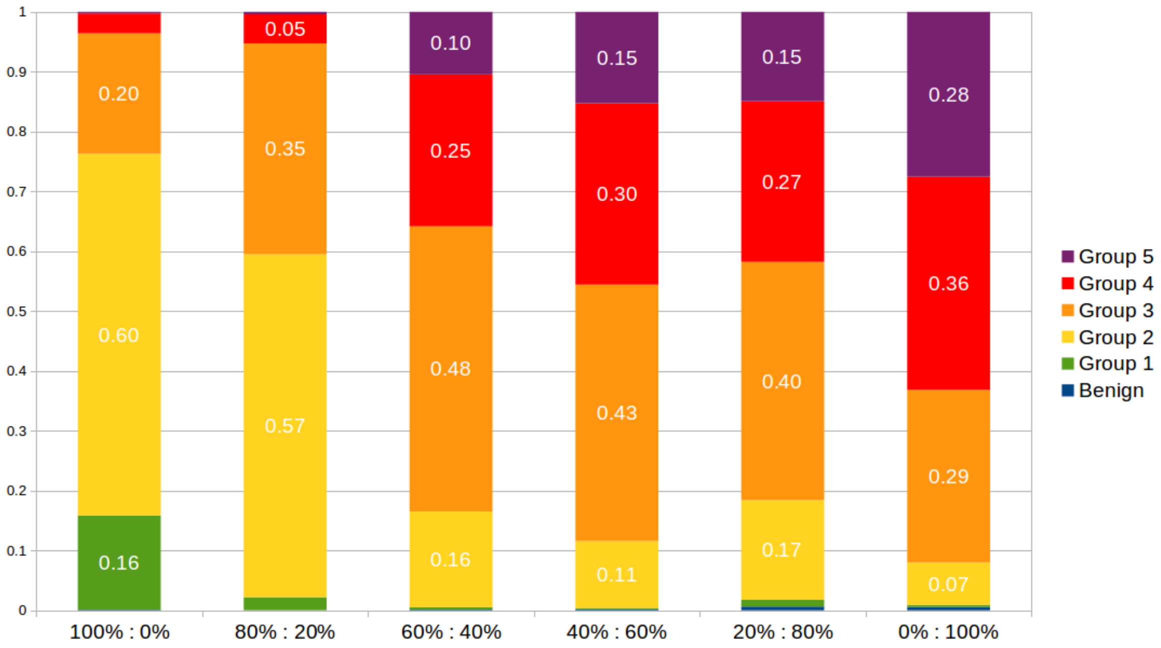

Supplementary Figure 3. Five diagrams showing the cumulative form of the second stage model outputs on the synthetic WSIs generated according to the combination ratio of five different WSI pairs, each with Gleason score 3+3 and 4+4. The first percentage is of WSI with 3+3 and the second with 4+4. For example, '80% : 20%' designates the synthetic WSI combining 80% area of WSI with 3+3 and 20% area of WSI with 4+4 (See Supplementary Figure 4).

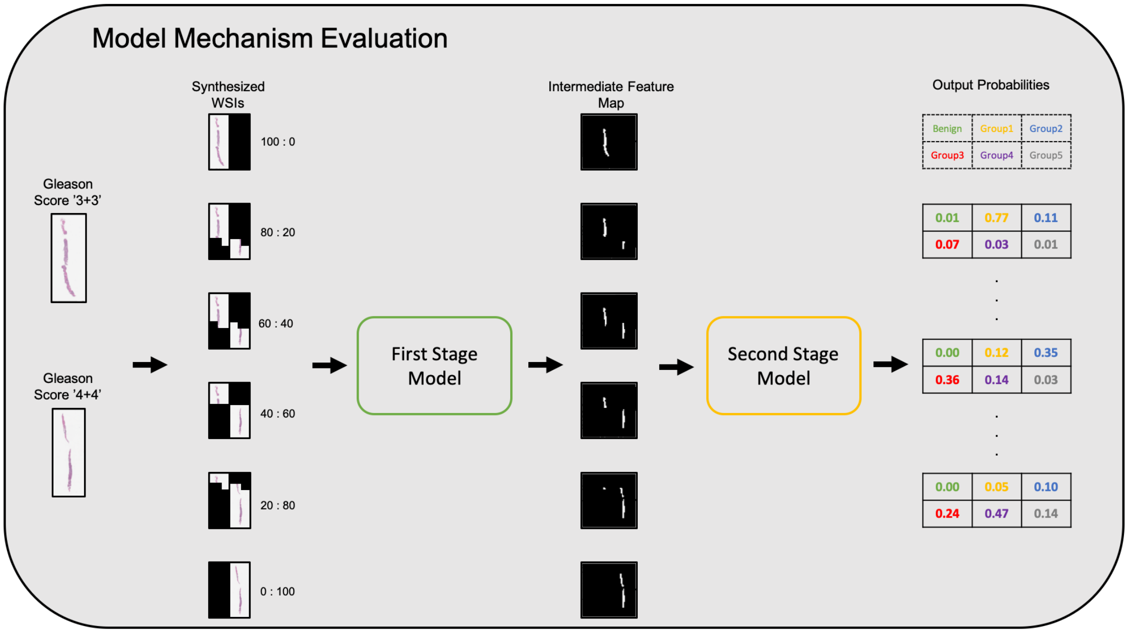

Supplementary Figure 4. Experiment workflow of second stage model mechanism evaluation. Given a pair of WSIs, six virtual WSIs were synthesized according to the predefined combination ratio values, which are then fed into the first stage model to generate feature maps. The second stage model was applied to these feature maps to obtain output values for each synthetic WSI.

Supplementary Table 1. Architecture diagram of the second stage model. First, five consecutive convolution layers are applied to compress the channels from 1,024 to 128. Then, 16 residual blocks are applied to generate a 1024 × 4 × 4 tensor. The global average pooling and linear softmax layer convert this tensor into the grade group probabilities output.

Layers	Stride	Output Shape
Convolution 2D (1)	1	512 × 64 × 64
Convolution 2D (2)	1	384 × 64 × 64
Convolution 2D (3)	1	256 × 64 × 64
Convolution 2D (4)	1	192 × 64 × 64
Convolution 2D (5)	1	128 × 64 × 64
Residual Block (1)	2	128 × 32 × 32
Residual Block (2)	1	128 × 32 × 32
Residual Block (3)	1	128 × 32 × 32
Residual Block (4)	1	128 × 32 × 32
Residual Block (5)	1	128 × 32 × 32
Residual Block (6)	2	256 × 16 × 16
Residual Block (7)	1	256 × 16 × 16
Residual Block (8)	1	256 × 16 × 16
Residual Block (9)	1	256 × 16 × 16
Residual Block (10)	1	256 × 16 × 16
Residual Block (11)	2	512 × 8 × 8
Residual Block (12)	1	512 × 8 × 8
Residual Block (13)	1	512 × 8 × 8
Residual Block (14)	1	512 × 8 × 8
Residual Block (15)	1	512 × 8 × 8
Residual Block (16)	2	1024 × 4 × 4

Supplementary Table 2. Architecture diagram of the residual blocks used in the second stage model. Originally, the residual block output is a residual sum of the original input and the output of the two consecutive 3 × 3 convolution layers on the input (Stride 1). When the spatial size resolution of the output should be reduced by 2, an additional 1 × 1 convolution layer of stride 2 is applied to the input before the residual sum is computed (Stride 2).

<table>
<tr><th colspan="2">Stride 1</th><th colspan="2">Stride 2</th></tr>
<tr><td>Convolution 2D
(3 × 3 kernel, stride 1)</td><td rowspan="2">Original Input</td><td>Convolution 2D
(3 × 3 kernel, stride 2)</td><td rowspan="2">Convolution 2D
(1 × 1 kernel, stride 2)</td></tr>
<tr><td>Convolution 2D
(3 × 3 kernel, stride 1)</td><td>Convolution 2D
(3 × 3 kernel, stride 1)</td></tr>
</table>
